# Frequency and trend analyses of annual peak discharges in the Lower Mekong Basin

**DOI:** 10.1016/j.heliyon.2023.e19690

**Published:** 2023-09-01

**Authors:** Uttam Pawar, Sophal Try, Nitin Muttil, Upaka Rathnayake, Worawit Suppawimut

**Affiliations:** a Department of Geography, HPT Arts and RYK Science College, Nashik, 422 005, Maharashtra, India; b Disaster Prevention Research Institute, Kyoto University, Kyoto, 611-0011, Japan; c College of Sport, Health and Engineering, Victoria University, P.O. Box 14428, Melbourne, VIC, 8001, Australia; d Department of Civil Engineering and Construction, Faculty of Engineering and Design, Atlantic Technological University, Sligo, F91 YW50, Ireland; e Department of Geography, Chiang Mai Rajabhat University, Tambon Chang Puak, Maung, Chiangmai, 50300, Thailand

**Keywords:** Lower Mekong Basin, Annual maximum series, Trend analysis, Goodness-of-fit, Flood-frequency analysis

## Abstract

The effectiveness of annual peak discharges under the anthropogenic impact and climate change has significance for disaster management and planning. Therefore, an attempt has been made to study the trend of annual maximum series (AMS) discharges and flood frequency in the Lower Mekong Basin (LMB). The AMS data of five stations in the LMB were procured from the Mekong River Commission for analyses of trends of the AMS and flood frequency. The Mann-Kendall test showed a significant decrease in the magnitude of annual peak floods for all the discharge gauging sites in the LMB. Likewise, the analysis of the annual discharge departure from the mean reveals noteworthy variations and departure (positive and negative) in the annual peak discharges. The goodness-of-fit (GoF) tests showed that Log-Pearson Type-III (LP-III) is the best distribution for AMS of the Mekong River than Gumbel Extreme Value Type-I (GEVI). Therefore, predicted discharges for different return periods and predicted recurrence intervals for average annual discharges (*Q*_*m*_), large floods (*Q*_*lf*_), and maximum annual peak discharge during the recording period (*Q*_max_) by LP-III are trustworthy. The flood frequency curve specified that all the observed discharges were fairly on the best-fitted line and falls between upper and lower confidence limits. Inclusively, the results of the trend in annual peak discharges and flood frequency are consistent and can be used for water management, controlling flood disasters, and flood planning in the LMB.

## 1 Introduction

Floods associated with heavy rainfall events over a river basin are the most recurrent, catastrophic, and widespread natural hazards throughout the world that causes massive loss of lives and the economy [1,2]. Annual inundating is a noteworthy hydrometeorological feature of the Lower Mekong Basin (LMB). Although annual peaks of floods become lesser in magnitude, there was an increase in loss of lives, structure, and economy in the Mekong region from 1984 to 2017 because of the continued increase in population and economic growth [3,4]. According to the Mekong River Commission, average annual economic losses due to floods in the LMB are about US$ 60–70 million [5]. Extreme flood events showed an increase in the magnitude of flood, and inundation area in the Mekong Basin (MB) under the climate change [6]. Severe floods in the MB were reported in the years 1924, 1929, 1939, 1940, 1941, 1961, 1966, 1971, 1978, 2000, 2008, and 2011 [7,8]. According to Cosslett and Cosslett [9], the assessed monetary damages of above US$ 200 million because of the historic flood of 2000 show high vulnerability to extreme floods in the Mekong Delta region. Nguyen et al. [10] mentioned that 11 million populations in 610 sub-districts were affected by the 2000 flood, out of which 4.5 million populations in the 77 communes were maximum suffered where inundation stages topped above 3 m. Shrestha et al. [11] reported agricultural damages of 155.10 million US$ in the 2000 flood and 123.40 million US$ in the 2006 flood. Further, 402,940 ha of agricultural areas in the Cambodian floodplain were affected in the 2000 flood [12] and 400,000 ha by the 2011 flood [13]. Frequent flooding is another severe disaster after typhoons in Vietnam that causes damage and mortality [14].

An assessment of climate change and identification of hydro-climatic trends have great importance in various disciplines such as hydrology, environment, and atmospheric science [15]. Irannezhad and Liu [16] examined a significantly increasing trend of wet days in the Lancang-Mekong Basin (1952–2015). Recently, Liu et al. [17] observed a significant rise in extreme rain in the LMB and a significant decrease in extreme rainfall in the Upper Mekong Basin (UMB). Besides, changes in the magnitude of extreme rainfall are significantly higher in the LMB as compared to the UMB which causes a higher possibility of flooding in the LMB. It is crucial to understand the hydrological trends of the Lancang-Mekong Basin. According to Yue and Wang [18], trend analysis is the most suitable and effective technique to detect changes in hydrological variables such as discharge and rainfall as it provides valuable information on the future change (trend) in the hydrometeorological variables. Cigizoglu et al. [19] applied the Mann-Kendall test and parametric *t*-test to notice trends in low, mean, and high streamflows of the selected river in Turkey and noted a declining trend except at a few stations. Burn et al. [20] analyzed trends in extreme hydrological events from 1957 to 2006 based on records of 68 discharge stations located in diverse hydrological conditions in Canada and documented that annual peak flows are generally becoming lesser and earlier.

Flood frequency analysis (FFA) has an importance in overcoming the hazardous effects of floods and hazard assessment and planning [21]. Moreover, FFA is a fundamental technique to comprehend the extent of floods required for a precise estimation of probable floods for the construction of hydraulic structures and flood management [22,23]. Globally, FFA is a commonly applied method for estimating design flood [24]. At-site FFA is the utmost direct approximation of design flood amongst numerous FFA techniques [25]. The precision of an estimated magnitude and return period significantly governed by the availability of continuous and long-term observed peak discharge data [26,27]. However, short annual peak discharge archives are the main limitations of the FFA [[28], [29], [30]]. To estimate precise and trustworthy discharges for various return periods, a minimum of 30 years of discharge data in continuous years can be sufficient [31,32].

The magnitude and recurrence interval of floods can be assessed by several flood frequency analysis (FFA) methods that usually comprise Generalized Extreme Value, Log-normal, Log-Pearson Type-III (LP-III) [33], and Gumbel Extreme Value Type-I (GEVI) [34,35]. However, it is very challenging to select an appropriate technique for FFA [[36], [37], [38], [39]]. The Gumbel distribution is appropriate for the prediction of the recurrence interval and magnitude of rainfall, and flood based on a low sample size while Log-normal gives good results for long-term stream flows [40,41]. According to Millington et al. [42], Generalized Extreme Value is the best-fit for the upper Thames watershed in the United Kingdom. Besides, the LP-III is one of the most frequently used in hydrology for the FFA [43]. The United State Water Resources Council [44] has recommended LP-III for the FFA of the rivers in the United States. Moreover, the LP-III was applied to the Australian rivers [45,46]. The Institute of Engineers Australia [47] also suggested that LP-III distribution is the most appropriate for the FFA of the Australian rivers. Pekarova et al. [48] recommend the LP-III as the most suitable distribution for FFA of the Danube River at Bratislava, Slovakia. In India, many investigators have used the GEVI and LP-III distributions for the FFA of monsoon-dominated rivers such as the Tapi River [49], Mahi River [32], Jhelum River [50], Rapti River [51], Narmada River [27], and the Krishna River [52].

There are very few studies on on-site FFA based on long-term annual maximum series (AMS) data from various discharge sites in the Mekong Basin. Recently, Thoummalangsy et al. [53] used only Gumbel probability distributions for FFA of the Xe Bangfai River based on 21 years (1994–2014) AMS data and computed discharges for various return periods. Kim et al. [54] studied the FFA of the LMB based on 30 years (1989–2019) discharge data of the Kampong Cham gauging station using LP-III, Log-normal, Normal, and GEVI distributions. The goodness-of-fit (GoF) investigation showed that LP-III is the most suitable for the FFA of the LMB (Cambodia). Therefore, the main objectives of this research are to detect a long-term trend in annual peak discharges and to find the best-fit distribution for the FFA of the Mekong River.

## 2 Study areas

**Fig. 1:**
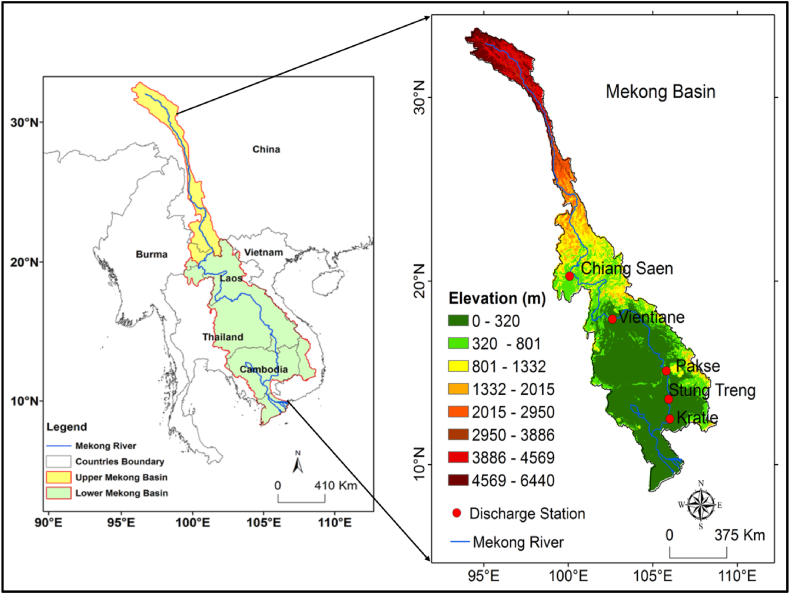
Location of the study area and discharge station in the Mekong Basin.

**Fig. 2:**
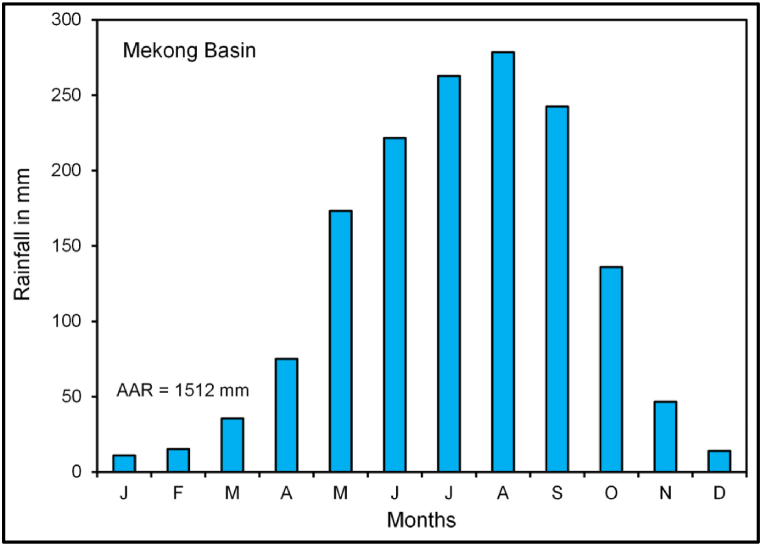
Average monthly rainfall distribution in the Mekong Basin.

The Mekong Basin is the biggest in Southeast Asia with a 795,000 km^2^ catchment area. It occupies areas of China, Myanmar, Thailand, Lao PDR, Cambodia, and Vietnam (refer to Fig. 1). The basin is classified as the UMB (24% of the total catchment area) and LMB (76% of the total catchment area) [55]. The Mekong River originates in the Tibetan highlands at an elevation of over 5244 m s l. and joins the South China Sea. It has approximately 4, 900 km in length. It is the 10th major river in the world in terms of average annual discharge (approximately 475 cubic kilometers) [56]. The Mekong Basin has a monsoon climate. About 90% of the annual precipitation was recorded between May and October [57]. The average annual rainfall of the Mekong Basin is 1512 mm (1950–2016). August is the rainiest month in the Mekong Basin (Fig. 2). Nevertheless, it varies between 300 mm to more than 3, 000 mm in the basin [[58], [59], [60]]. The LMB receives maximum rain from June to October and the extreme stream flows occur in September and October [55]. According to Chen et al. [61], rainfall variability in the MRB is because of elevation, land-atmosphere interactions, different weather systems, and climate conditions.

## 3 Data and methodology

### 3.1 Data used for the analysis

A hydrologist regularly uses the FFA to compute discharges for various return periods based on AMS data. Therefore, observed AMS discharge data were acquired from the Mekong River Commission for the five discharge stations along the mainstream namely, Chiang Saen, Vientiane, Pakse, Stung Treng, and Kratie. The AMS data length varies from 61 to 111 years. In addition, rainfall data were used to study the monthly average rainfall of the Mekong Basin. The hydrological parameters of the Mekong River at various discharge stations were represented in Table 1.

**Table 1:** Hydrological parameters of the Mekong River.

Discharge Stations	Country	Data length (Years)	Length of the river (km)	Upstream catchment area (km^2^)	Elevation (m)	Q_max_ (m^3^/s) (Year)
Chiang Saen	Thailand	1960–2020 (61)	1730	207,488	378	23500 (1966)
Vientiane	Lao PDR	1923–2016 (94)	2420	326,392	168	26633 (2008)
Pakse	Lao PDR	1923–2020 (98)	3000	574,462	95	57800 (1978)
Stung Treng	Cambodia	1910–2020 (111)	3200	661,700	47	78093 (1939)
Kratie	Cambodia	1924–2020 (97)	3300	677,297	14	76100 (1978)

### 3.2 Methodology

The inundation features of the Mekong River were determined using basic statistic methods specifically, mean, standard deviation (σ), coefficient of variation (Cv), and coefficient of skewness (Cs). In addition, serial autocorrelation was checked for trend analysis of AMS. The Mann-Kendall test [62,63] and Sen's Slope test [64] were applied to identify changes in discharges. A brief methodology was mentioned as follows. A detailed methodology can be found in Pawar and Upaka [65] and Pawar [66]. GoF tests for example Anderson-Darling (AD) test, Kolmogorov-Smirnov (KS), and Chi-Square test ($x^{2}$) were used to choose the best-fit probability distribution at every site on the Mekong River. To find out the best-fit probability distribution at-a-site ranks were allotted to the GEVI and LP-III based on the GoF test results. The 1st rank denoted acceptance of the distribution and the rank 2nd specifies rejection of the distribution.

#### 3.2.1 Serial autocorrelation

Pre-whitening is a method to sort the interrelated data before using nonparametric tests. Consequently, the annual maximum discharge data were tested using serial autocorrelation. The serial autocorrelation was confirmed using the PAST 4.03 version of the software, and correlograms were plotted for AMS of the Mekong Basin at varying time lags.

#### 3.2.2 The Mann-Kendall test

The trends in the yearly maximum peak discharges were checked by using the following Eq. (1).

(1) $$S=\sum_{i=1}^{n-1}\sum_{j=i+1}^{n}sgn\left(X_{j}-X_{i}\right)$$

where n is the number of data points; X_i_ and X_j_ are the data values in the time series; i and j (j > i), respectively, and sgn (X_j_– X_i_) is the sign function as Eq. (2).

(2) $$sgn\left(X_{j}-X_{i}\right)=\left\{ \begin{array}{c} +1\;if\;\left(X_{j}-X_{i}\right)>0\\ 0\;if\;\left(X_{J}-X_{i}\right)=0\\ -1\;if\;\left(X_{j}-X_{i}\right)<0 \end{array}\right.$$

when n≥10, S becomes approximately normal distribution with mean = 0 and variance as mentioned in Eq. (3).

(3) $${\sigma_{s}}^{2}=\frac{1}{18}\left[n\;\left(n-1\right)\left(2n+5\right)-\Sigma_{t}\;t\left(t-1\right)\left(2t+5\right)\right]$$

where t is the extent of any given tie and $\Sigma_{t}$ indicates the summation of all ties. The value of $Z_{c}$ is computed using Eq. (4).

(4) $$Z_{c}=\left\{ \begin{array}{c} \frac{S-1}{\sigma_{s}},S>0\\ 0,S=0\\ \frac{\left(S+1\right)}{\sigma_{s}},S<0 \end{array}\right.$$

where Z is the standard normal variate; Positive (negative) values of Z indicate increasing (decreasing) trends. A null hypothesis is rejected when |Z| > Z_1_-α_/2_, and a noteworthy trend occurs in the time series. All the results are tested at α = 0.05 (Z = ±1.96) significance level.

#### 3.2.3 Sen's slope test

Sen's slope method was used to find out the magnitude of trend/slope (β) by using the Eqs. ((5), (6)).

(5) $$Q_{i}=\frac{X_{j}-X_{k}}{j-k},i=1,2,\ldots \ldots .\;N$$

where X_j_ and X_k_ are values in series at time j and k (j > k), respectively.

The median of these N values of Q_i_ is Sen's estimator of the slope. If there is a single datum in each period, then N = n(n-1)/2 where n is the number of periods.

Nevertheless, if the number of values every year is many, then N < n(n-1)/2 where n is the total number of observations. First N values were ranked from smallest to biggest. Then, the median of slope (β) is computed as:

(6) $$\beta =\left\{ \begin{array}{c} \frac{Q_{n}+1}{2}\;if\;N\;is\;odd\\ \frac{1}{2}\left(\frac{Q_{n}}{2}+\frac{Q_{n}+2}{2}\right)\;if\;N\;is\;even \end{array}\right.$$

#### 3.2.4 Annual maximum peak flood magnitude change (%)

Annual maximum peak flood magnitude change (%) was calculated by Eq. (7), [67].

(7) $$Percent\;change\;\left(\%\right)=\frac{\beta \;X\;length\;of\;year}{mean}\;x\;100$$

#### 3.2.5 Gumbel extreme value type I probability distribution

Globally, the GEVI [34,35] is a widely used probability distribution in FFA. Accordingly, discharges for different return periods *(Q*_*2*_*, Q*_*5*_*, Q*_*10*_*, Q*_*25*_*, Q*_*50*_*, Q*_*100,*_ *Q*_*200,*_ and *Q*_*500*_ years) were computed by using the Eqs. ((8), (9), (10)).

(8) $$\bar{Q}=\frac{\sum Q}{n}$$

(9) $$\sigma Q=\sqrt{\frac{\sum {\left(Q-\bar{Q}\right)}^{2}}{n-1}}$$

(10) $$Q_{T}=\bar{Q}+\left(K_{\left(T\right)}*\sigma Q\right)$$

where *Q*_*T*_ is a discharge of a given return period; $\bar{Q}$ is a mean annual peak discharge; *σQ* is a standard deviation of AMS and *K*_*(T)*_ is a frequency factor which is the function of the return period T. Moreover, the recurrence interval was projected for mean annual peak discharge (*Q*_*m*_), large flood (*Q*_*lf*_) (all floods above mean plus one standard deviation (> $\bar{Q}$ +1σ)) [49], and annual peak discharge observed during the gauge period (*Q*_max_) at every site. The *F(X)* value was computed using Eqs. ((11), (12), (13)).

(11) $$F\left(X\right)=e^{ˆ}({-e}^{ˆ}\left(-b\left(x-a\right))\right)$$

where *F(X)* is the probability of an annual maximum *Q ≤ X* and *a* and *b* are two parameters related to the moments of the population of *Q* values. The parameters *a* and *b* were determined by using Equations (12), (13)).

(12) $$b=\frac{\pi }{\sigma Q\sqrt{6}}$$

(13) $$a=\bar{Q}-\frac{\gamma }{b};\;\left(\gamma =0.5772\right)$$

Finally, the return periods for desired discharges (Qm, Qlf, and Qmax) were calculated by using Eq. (14).

(14) $$T=\frac{1}{1-F\left(X\right)}$$

where *F(X)* is the possibility of an annual maximum flood.

#### 3.2.6 Log-Pearson type-III probability distribution

The first stage in the FFA using LP-III is to convert AMS values into logarithms and compute the mean, standard deviation (*σ*), and coefficient of skewness (*Cs*). Accordingly, flood discharges were predicted for various return periods based on logarithms of AMS using the following Eqs. ((15), (16), (17), (18), (19)).

(15) $$\bar{logQ}=\frac{\Sigma logQ}{n}$$

(16) $$\sigma \;\log\;Q=\sqrt{\frac{\sum {\left(logQ-\bar{logQ}\right)}^{2}}{n-1}}$$

(17) $$C_{s}=\frac{n*\sum {\left(logQ-\bar{logQ}\right)}^{3}}{{\left(n-1\right)*\left(n-2\right)*\left(\sigma logQ\right)}^{3}}$$

(18) $${logQ}_{T}=\bar{logQ}+\left(K_{\left(T\right)}*\sigma_{\log}Q\right)$$

(19) $$Q_{T}=Antilog\left(\log\;Q_{T}\right)$$

where *logQ*_*T*_ is the base 10 logarithms of the discharge of desired return period; $\bar{logQ}$ is a mean of the logarithms of AMS; *σ*_*log*_*Q* is a standard deviation of the logarithms of AMS and *K*_*(T)*_ is a function of the return period (T) and skewness coefficient (Cs). The *K*_*(T)*_ values were obtained from tables given in the book on Hydrology [68,69].

#### 3.2.7 Confidence limit for LP-III curve

The 95% confidence limits were calculated as mentioned in the United State Water Resources Council [70] Bulletin 17B and shown on the flood frequency curve. Eq. (20), (21) were used to obtain confidence limits for the LP-III distribution.

(20) $$X_{CI,U}=\bar{X_{1}}+S_{1}\left(K_{CI,U}\right)$$

(21) $$X_{CI,L}=\bar{X_{1}}+S_{1}\left(K_{CI,L}\right)$$

where, *X*_*CI, U*_ is the logarithmic upper confidence limit; *X*_*CI, L*_ is the logarithmic lower confidence limit; $\bar{X_{1}}$ is the logarithmic peak flow mean and *S*_*1*_ is the logarithmic peak flow standard deviation. The values of the *K*_*CI, U,*_ and *K*_*CI, L*_ were computed using the Eqs. ((22), (23)).

(22) $$K_{CI,U}=\frac{K_{LP,T}+\sqrt{K_{LP,T}^{2}-ab}}{a}$$

(23) $$K_{CI,L}=\frac{K_{LP,T}-\sqrt{K_{LP,T}^{2}-ab}}{a}$$

The values of ‘a’ and ‘b’ were computed using following Eqs. ((24), (25)).

(24) $$a=1-\frac{Z_{C}^{2}}{2\;\left(n-1\right)}$$

(25) $$b=K_{LP,T}^{2}-\frac{Z_{C}^{2}}{n}$$

where *n* is the record length; *K*_*LP*,__*T*_ is the *K*_*(T)*_ value of LP-III, as a function of return period and skew coefficient and *Z*_C_ is the standard normal deviation for 95% confidence limit (Z_C_ = 1.64485).

## 4 Results

### 4.1 Serial autocorrelation analysis

**Fig. 3:**
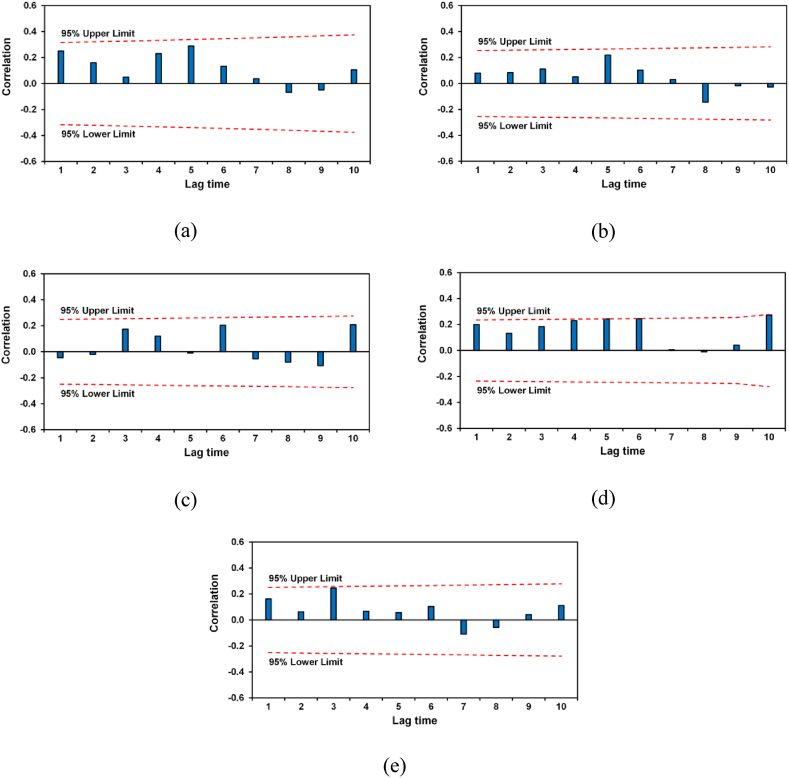
Plots of the serial autocorrelation: (a) Chiang Saen Station; (b) Vientiane Station; (c) Pakse Station; (d) Stung Treng Station; (e) Kratie Station.

A serial autocorrelation in the AMS data of the Mekong River was checked and the correlogram was derived based on the results of serial autocorrelation analysis. The correlogram indicates that annual maximum discharges were between the confidence limits (Fig. 3). This suggests that there is no serial autocorrelation in the annual peak discharge series of Chiang Saen, Vientiane, Pakse, Stung Treng, and Kratie stations [Fig. 3(a-e)]. Therefore, original AMS discharge data were directly used to understand the hydrological trend in the LMB.

### 4.2 Hydrological trend analysis

The outcomes of the Mann-Kendall test, Sen's Slope test, and percent change were summarized in Table 2. A noteworthy decreasing trend in the AMS at the 0.05 level was observed for the Chiang Saen (Z = −3.62), Vientiane (Z = −2.86), and Stung Treng (Z = −4.36), and Kratie (Z = −2.85) and for the Pakse station (Z = −1.96) at the 0.10 level of significance (Table 2). The Sen's Slope test and percentage change analysis showed that the magnitude of the annual peak discharges varied between −12% (at Pakse) and −47% (at Chiang Saen) during the recording period of the respective station. Delgado et al. [71] observed an increasing (decreasing) probability of extreme floods (average floods) after the 1950s in the Mekong River. According to Lu and Siew [72], the construction of the dams in the UMB could have had significant influences on the flood regime in terms of flood magnitude and variability at downstream stations like Kratie during the flood season. Li et al. [73] observed a decrease in maximum streamflows at the Chiang Saen because of the reduction of streamflows following the completion of dams (Xiaowan and Nuozhadu dams) upstream of the Mekong River. Further, they stated that flood duration, magnitude, and maximum water level were reduced throughout the Mekong Basin due to dam construction. According to Wang et al. [74] construction of dams can efficiently reduce flood magnitude and frequency in the Lancang-Mekong Basin. On the contrary, Li and He [75] observed that the water level pattern at the upstream dam was similar before and after the completion of the dam and highlighted the influence of climatic factors on the upper Mekong water level fluctuations. Wu et al. [76] observed a slight increase in the annual precipitation from 1960 to 2000 and decreasing trend after 2000 in the UMB. Likewise, a substantial reduction in annual rainfall over western Thailand (LMB) during 1961–2007 was noted by Artlert and Chaleeraktrakoon [77]. Irannezhad et al. [78] observed a significant increase in the wet days (R1mm) in the Lancang-Mekong Basin. Moreover, consecutive wet days showed a noteworthy increase in the east, south, and northwestern part of the Mekong Basin, whereas in the western part of Mekong Basin and the north of the Lancang Basin consecutive wet days denoted a significant decrease [78). Therefore, the probable impact of climate change on flood dynamics in the Lancang-Mekong Basin is a topic of deep consideration [79].

**Table 2:** Result of change detection in annual peak series on the Mekong River.

Discharge Stations	Record Length (Years)	Mann Kendall (Z)	Sen's Slope (***β***)	Change (%)	Critical values
Chiang Saen	61	−3.62	−76.30	−47	2.000*
Vientiane	94	−2.86	−33.33	−19	1.987*
Pakse	98	−1.96	−43.95	−12	1.660**
Stung Treng	111	−4.36	−122.22	−26	1.984*
Kratie	97	−2.85	−82.64	−18	1.984*

* = Significant at 0.05 level; ** = Significant at 0.10 level; See Fig. 1 for location of sites.

### 4.3 Annual peak discharge departure from mean

**Fig. 4:**
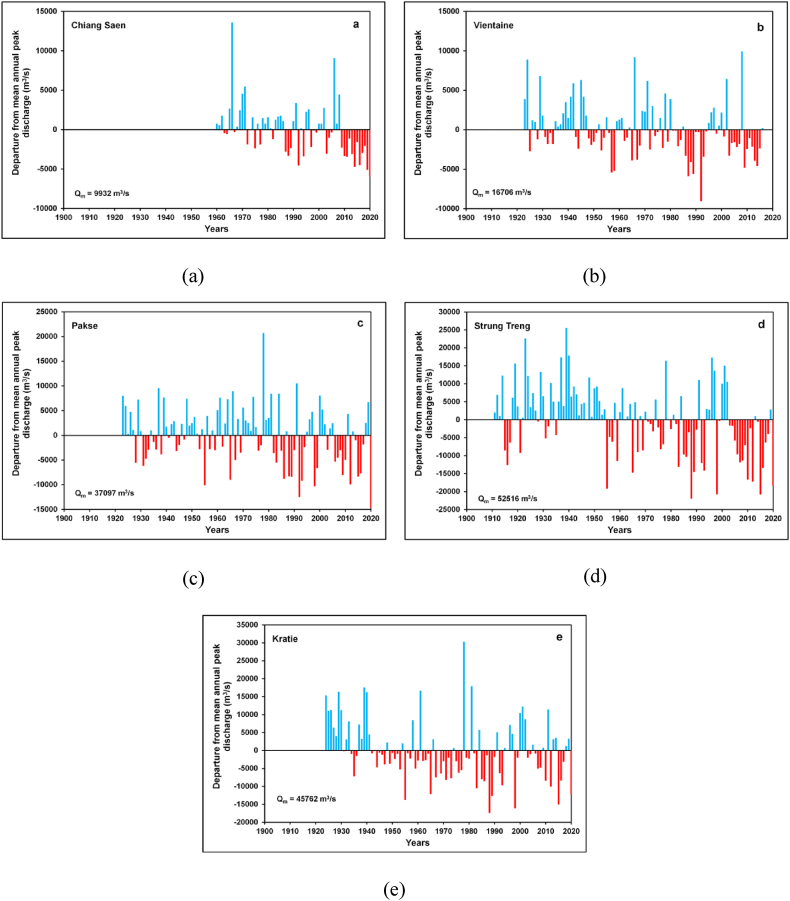
Plots of discharge departure from mean annual peak flows (a) Chiang Saen Station; (b) Vientiane Station; (c) Pakse Station; (d) Stung Treng Station; (e) Kratie Station.

Fig. 4(a–e) showed noteworthy positive and negative departures in annual peak discharges from the average annual peak discharge of the respective stations. The maximum positive departure from mean annual peak discharge was observed at the Chiang Saen (137%) and Vientiane (59%) stations in the year 1966 [Fig. 4(a and b)]. According to Adamson et al. [80], very high positive deviations in annual peak discharges are significantly associated with cyclones and severe tropical storms. In 1966, the largest discharges were recorded on the Mekong River because of the tropical storm Phyllis that hit the UMB and produced floods in the LMB mainly at the floodplain of the Cambodia and the Vietnamese Mekong Delta [80]. On the other side, Chiang Saen's annual peak discharge (4, 015 m^3^/s) was very less (−60%) than the mean annual discharge (9, 932 m^3^/s) of the station in the year 2020 (Fig. 4(a)). Likewise, the annual discharge (7, 650 m^3^/s) recorded in 1992 at the Vientiane was 54% lower than the mean annual peak discharge (16, 706 m^3^/s) of the station [Fig. 4(b)]. Moreover, at the Pakse (56%) and Kratie (82%), annual peak discharges were recorded much higher than the mean annual discharges of the respective stations in the year 1978 [Fig. 4(c, e)]. The largest annual peak discharge in the Mekong Basin recorded in 1939 at the Stung Treng (78,093 m^3^/s) was greater by about 42% than the mean annual peak discharge (30, 532 m^3^/s) of the Stung Treng during 1923–2020 [Fig. 4(d)]. In opposition, annual peak discharges at the Stung Treng (−42%) and the Kratie (−47%) were much below the mean annual discharges of the respective stations in the year 1988 [Fig. 4(d and e)]. Previous studies on the precipitation pattern over the Mekong Basin specified that annual rainfall over the UMB has reduced in recent times causing a rise in drought years in the Mekong Basin [76,81]. This affected the yearly peak discharges of the Mekong River in the LMB. Therefore, the analyses suggested that there are notable departures (positive and negative) in the annual flood discharges of the Mekong River.

### 4.4 Descriptive statistical analysis of annual maximum discharges

**Fig. 5:**
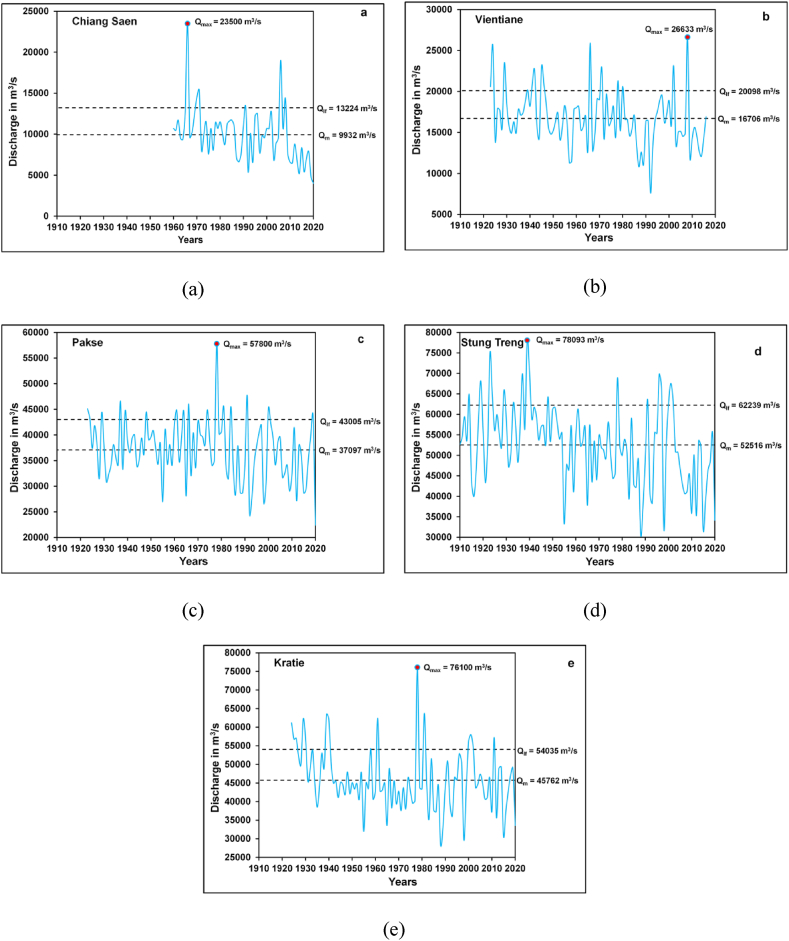
Time series plots of annual maximum discharges on the Mekong River: (a) Chiang Saen Station; (b) Vientiane Station; (c) Pakse Station; (d) Stung Treng Station; (e) Kratie Station.

The flood characteristics of the Mekong River were studied by using AMS discharge data and the results were shown in Table 3. The AMS data ranges between 61 years (Chiang Saen) and 111 years (Stung Treng). The mean annual discharges vary between 9932 m^3^/s (Chiang Saen) and 52,516 m^3^/s (Stung Treng). Furthermore, the lowest value of a large flood was noted for Chiang Saen (13, 224 m^3^/s) and the highest value of a large flood was observed for Stung Treng (62,239 m^3^/s) (Table 3). The *Q*_max_ was recorded in the Mekong Basin at the Stung Treng (78, 093 m^3^/s) in 1939. The *Q*_max_*/Q*_*m*_ ratio denotes that *Q*_max_ was about 2–3 times greater than *Q*_*m*_ in the Mekong Basin (Table 3). The values of *Cv* range between 0.16 (16%) and 0.33 (33%) which indicated that there are no remarkable variations in the yearly peak floods of the Mekong River. Besides, the maximum *C*_*s*_ value (1.34) for Chiang Saen specifies that few high-magnitude floods had occurred in the past whereas the lowest value of *C*_*s*_ (−0.05) observed for Stung Trend indicated some of the years (42 years) of low discharges than the mean discharge (*Q*_*m*_) of the Stung Treng in the last 111 years. To understand spatiotemporal variations in the peak discharges on the Mekong River, time series plots of annual maximum discharges at sites were developed for every station under review [Fig. 5(a-e)]. The time series plots showed some very high peaks of discharges at every station with interannual variations [Fig. 5(a-e)]. The extremely high flood discharges were considerably linked with the active and vigorous southwest monsoon conditions resulting from tropical cyclones such as 1939, 1966, 1976, and 2011 [82]. According to CostaCabral et al. [83] and Delgado et al. [84], the southwest monsoon rainfall significantly determined the flood regime of the Mekong River.

**Table 3:** Flood flow characteristics of the Mekong River.

Discharge Stations	N	Q_min_ (m^3^/s)	Q_m_ (m^3^/s)	Q_lf_ (m^3^/s)	Q_max_ (m^3^/s) (Year)	Q_max_/Q_m_	σ	C_v_	C_s_
Chiang Saen	61	4015	9932	13224	23500 (1966)	2.37	3292	0.33	1.34
Vientiane	94	7650	16706	20098	26633 (2008)	1.59	3392	0.20	0.59
Pakse	98	22399	37097	43005	57800 (1978)	1.56	5907	0.16	0.15
Stung Treng	111	30532	52516	62239	78093 (1939)	2.56	9723	0.19	−0.05
Kratie	97	28356	45762	54035	76100 (1978)	1.66	8273	0.18	0.72

### 4.5 Goodness-of-fit analysis

The most frequently used test of GoF such as the KS-test, AD-test, and *χ*^2^ –test were applied to AMS of discharges at sites on the Mekong River. All the results obtained by using Easyfit software were tested at a 95% confidence level. The orders were allotted to the GEVI and LP-III probability distribution based on their probability values. The rank 1st displays approval (best-fit) of the distribution method and the rank 2nd designates rejection of the distribution. The KS-test, AD-test, and *χ*^2^ -test showed the LP-III is the most suitable distribution for all discharge stations on the Mekong River under study (refer to Table 4).

**Table 4:** Goodness-of-fit test results of discharge stations on the Mekong River.

Discharge Stations	Kolmogorov-Smirnov (KS) Test				Anderson-Darling (AD) Test				Chi Square *χ*^2^ Test			
	GEVI	Rank	LP-III	Rank	GEVI	Rank	LP-III	Rank	GEVI	Rank	LP-III	Rank
Chiang Saen	0.105	2	0.064	1	2.879	2	0.654	1	13.001	2	3.172	1
Vientiane	0.104	2	0.062	1	3.004	2	1.017	1	13.286	2	5.464	1
Pakse	0.104	2	0.062	1	2.973	2	1.062	1	14.114	2	5.194	1
Stung Treng	0.104	2	0.062	1	3.414	2	1.051	1	15.229	2	5.276	1
Kratie	0.104	2	0.061	1	2.973	2	1.205	1	14.114	2	5.860	1

### 4.6 Estimation of discharges

**Fig. 6:**
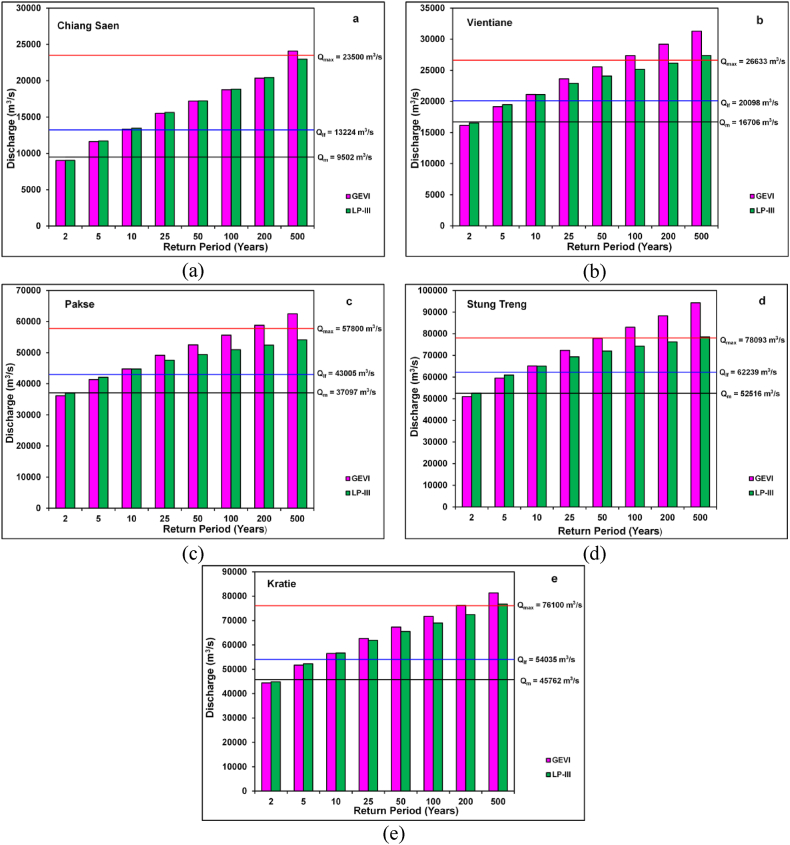
Plots of estimated discharges and return periods by Gumbel Extreme Value - I and Log-Pearson Type – III: (a) Chiang Saen Station; (b) Vientiane Station; (c) Pakse Station; (d) Stung Treng Station; (e) Kratie Station.

The discharges were predicted for various recurrence periods using GEVI and LP-III distribution (Table 5, Table 6). The relative analysis between GEVI and LP-III showed that discharges estimated by LP-III distribution were more accurate and very close to the observed Q_max_ at every station on the Mekong River. For instance, at Strung Treng, the estimated discharge by LP-III distribution for Q_500_ years return period was near to the recorded discharge (Q_max_ = 78, 093 m^3^/s) (Table 6). Likewise, the observed discharge at Kratie (76, 100 m^3^/s) was very close to the predicted discharge (76, 871 m^3^/s) for Q_500_ years return period (Table 6). Nevertheless, projected discharges for the Q_2_ return period were more or less very close to the mean annual discharges at every site under study (Table 3, Table 6). The expected discharges of different recurrence intervals were presented graphically [Fig. 6(a-e)]. These graphs specified that estimated flood flows for the Q_2_-year recurrence interval were the mean discharges. Nevertheless, predicted discharges for the Q_5_ and Q_10_ year recurrence interval were close to the large floods (Q_lf_).

**Table 5:** At-site estimated discharges (m^3^/s) for various return periods using Gumbel Extreme Value - I.

Discharge Stations	Q_2_	Q_5_	Q_10_	Q_25_	Q_50_	Q_100_	Q_200_	Q_500_
Chiang Saen	9405	12302	14212	16648	18524	20269	22047	24088
Vientiane	16163	19148	21116	23626	25559	27357	29189	31292
Pakse	36152	41351	44777	49149	52516	55647	58837	62499
Stung Treng	50960	59517	65156	72351	77893	83046	88297	94325
Kratie	44438	51719	56517	62639	67355	71739	76207	81336

**Table 6:** At-site estimated discharges (m^3^/s) for various return periods using Log-Pearson Type - III.

Discharge Stations	Q_2_	Q_5_	Q_10_	Q_25_	Q_50_	Q_100_	Q_200_	Q_500_
Chiang Saen	9493	12403	14221	16421	17997	19521	21025	22975
Vientiane	16536	19483	21107	22895	24079	25155	26150	27369
Pakse	37066	42865	45939	49224	51336	53213	54926	56977
Stung Treng	52582	60906	65094	69368	71999	74267	76251	78547
Kratie	44915	52268	56682	61873	65518	69021	72414	76871

### 4.7 Estimation of the return period

The GEVI distribution showed the return periods of 2.33 and 6.93 years for mean annual peak discharges and large floods for all stations on the Mekong River respectively. Leopold et al. [85] stated that rivers will equal or above the mean annual flood once every 2.33 years. Nevertheless, the recurrence interval of the Q_max_ varies from 52 years (for Stung Treng) to 351 years (for Chiang Saen) for the GEVI analysis. The GEVI analysis showed the highest return period (351 years) for the Q_max_ (23, 500 m^3^/s) observed at Chiang Saen. The return period of the Q_max_ (78, 093 m^3^/s) at the Stung Trend is 52 years as per the GEVI results (Table 7). On the other side, as per the best-fitted LP-III distribution, the recurrence interval of Q_m_ varied between 2.04 years (for Stung Treng) to 2.51 years (for Chiang Saen). The recurrence interval of Q_lf_ ranged between 6.56 years (for Stung Treng) and 9.94 years (for Vientiane) (Table 7). Nevertheless, the recurrence period of the Q_max_ fluctuates from 423 years (for Chiang Saen) to 850 years (for Pakse) (Table 7). The comparative analysis of the recurrence interval acquired based on the GEVI and LP-III showed that LP-III is most suitable distribution to all discharge stations under study. Therefore, LP-III distribution is the most appropriate to compute probable flood for the construction of hydraulic constructions and flood calamity planning and management in the Mekong Basin. An estimation of the recurrence period for large and extremely large floods is vital in flood-frequency analysis.

**Table 7:** Estimated return periods of Q_m_, Q_lf_ and Q_max_ for different sites on the Mekong River.

Discharge Stations	Q (m^3^/s)	Return period in years	Return period in years
		GEVI	LP-III
Chiang Saen	Q_m_ = 9932	2.33	2.51
	Q_lf_ = 13224	6.93	7.35
	Q_max_ = 23500	351	423
Vientiane	Q_m_ = 16706	2.33	2.19
	Q_lf_ = 20098	6.93	9.94
	Q_max_ = 26633	76	680
Pakse	Q_m_ = 37097	2.33	2.18
	Q_lf_ = 43005	6.93	7.21
	Q_max_ = 57800	160	850
Stung Treng	Q_m_ = 52516	2.33	2.04
	Q_lf_ = 62239	6.93	6.56
	Q_max_ = 78093	52	560
Kratie	Q_m_ = 45762	2.33	2.37
	Q_lf_ = 54035	6.93	7.05
	Q_max_ = 76100	196	547
*Q*_*m*_ = mean annual peak discharge; *Q*_*lf*_ = large flood; *Q*_max_ = maximum annual peak discharge: See Fig. 1 for location of sites.			

### 4.8 Flood-frequency curve analysis

**Fig. 7:**
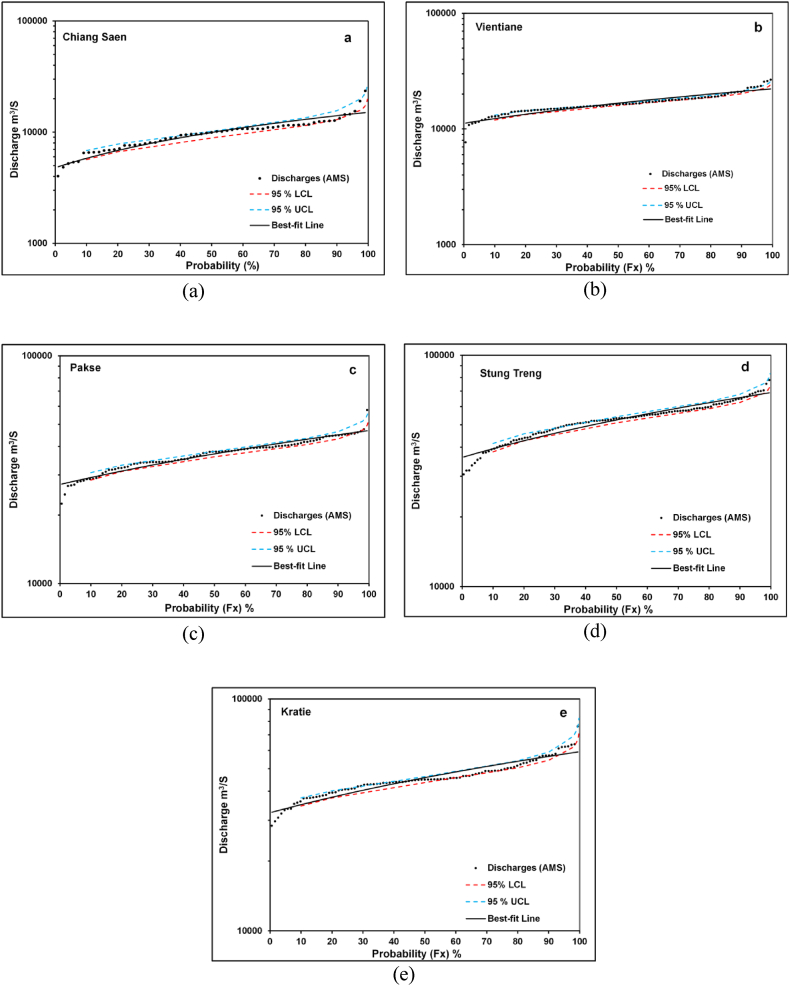
Flood frequency curves (a) Chiang Saen Station; (b) Vientiane Station; (c) Pakse Station; d) Stung Treng Station; (e) Kratie Station.

The observed discharges were plotted for all stations along the Mekong River by LP-III distribution which is the best-fitted to FFA of the Mekong River as per the GoF test. [Fig. 7(a-e)] showed that observed discharges are within the 95% confidence limits except for higher discharges (Vientiane) and lower tail discharges of all the sites. Besides, discharges are either on the best-fit line or close to it. Therefore, LP-III distribution is the most suitable for FFA of the Mekong River and to estimate discharges for various return period and to obtain the return periods for desired discharges [Fig. 7(a-e)]. This also strongly recommended that LP-III is the best-fit distribution for the FFA of the Mekong River. FFA has great significance for mapping flood-prone areas by a flood of a specified interval [86]. Chow et al. [23] stated that the flood frequency curve is used to design hydraulic structures such as dams and bridges. Dang et al. [87] dam development alone will reduce the submerged area in the Mekong Delta by 6% (3%) during wet (dry) years. Therefore, the FFA of the LMB has significance for flood-prone area mapping, construction of hydraulic structures, and managing and overcoming challenges in the LMB floodplain for better flood management.

## 5 Practical applications of the carried research work

This paper analyzed the flood characteristics in the LMB using statistical approaches. The estimation of frequency and trend analyses of annual peak discharges in the LMB can provide a further understanding of how often historical floods occur. The analysis was conducted at five hydrological stations along the mainstream of the Mekong River Basin: Chiang Saen, Vientiane, Pakse, Stung Treng, and Kratie. This study estimated the river flow at different return periods and predicted the recurrence interval for mean annual flow, large flow, and maximum annual peak flow during the historical recorded periods. This information could serve as principal information to be implemented in climate change adaptation, water resources management and planning, natural disaster prevention and adaptation strategies, and design of water-related infrastructures, including hydropower dams, irrigation reservoirs, and other flood prevention infrastructures in the LMB and other flood-prone river basins in Asia.

## 6 Conclusions

An analysis of flood magnitude, recurrence period, and trends in annual peak discharges in the LMB is the central aspect of the research. Accordingly, the annual peak discharge data of the five discharge stations located in the LMB were acquired from the Mekong River Commission and analyzed to identify the most suitable distribution amongst GEVI and LP-III. The variability of peak discharges showed variation with one or two high flood peaks throughout the gauge period in the LMB. *Q*_max_*/Q*_*m*_ ratio indicates that observed maximum annual peak discharges *(Q*_max_*)* are 2–3 times higher than mean discharges. The Mann-Kendall test showed a noteworthy decreasing trend in the AMS for Chiang Saen, Vientiane, Stung Treng, and Kratie at the 0.05 level of significance. However, a significant decrease in the AMS was observed for the Pakse station at the significance level of 0.10. The plots of the peak discharge departure from the mean displayed distinguished positive and negative deviations in the AMS of the Mekong River. The trend analysis specified that the declining trends in the peak discharges need a comprehensive understanding by considering anthropogenic and climatic aspects. It is the most important while executing flood-frequency analysis for flood disaster management and designing flood-protection infrastructures with a long design life. The GoF test analysis confirmed that the LP-III distribution is the most suitable distribution than the GEVI for flood-frequency analysis of the Mekong River. The flood frequency curves denoted that estimated discharges for different recurrence periods are either close to or on the fitted line. Therefore, the analysis showed that LP-III is the best-fitted for flood-frequency studies in the LMB.

## Author contribution statement

Uttam Pawar: Conceived and designed the experiments; Performed the experiments; Analyzed and interpreted the data; Contributed reagents, materials, analysis tools or data; Wrote the paper.

Sophal Try: Performed the experiments; Analyzed and interpreted the data.

Nitin Muttil: Worawit Suppawimut: Contributed reagents, materials, analysis tools or data.

Upaka Rathnayake: Conceived and designed the experiments; Wrote the paper.

## Data availability statement

Data will be made available on request.

## Declaration of competing interest

The authors declare that they have no known competing financial interests or personal relationships that could have appeared to influence the work reported in this paper.
